# The Rubber Question

**Published:** 1867-08

**Authors:** 


### THE RUBBER QUESTION.

We have just received from the publishers, some copies of the argument made by Geo. Ticknor Curtiss, on behalf of the defence, in the case of the Goodyear Dental Vulcanite Company, *versus* T. G. Waite, of New York; made before Justice Nelson, in the U. S. Circuit Court for the Southern District of New York, in that city, on the 5th, 6th and 7th of June last, in which all the legal points involved in this controversy are fully set forth, and ably argued. It is a most masterly production. Those who wish to become thoroughly posted, can do so, by obtaining and examining this argument, better than by any or all other means now available. We will take pleasure in forwarding it to any who may wish it, upon the receipt of the publisher's price, $1 50, or it may be obtained of S. D. Law, Esq., No. 25 Pine street, New York.

